# Verifying Urdu news authenticity using deep learning with concatenated BERT and GloVe embedding

**DOI:** 10.1038/s41598-026-36771-0

**Published:** 2026-02-05

**Authors:** Asif Feroz, Waseem Abbasi, Muhammad Zeeshan Babar, Abeer Aljohani

**Affiliations:** 1 Department of Computer Science & IT, Superior University, Sargodha Campus, Sargodha, 40100 Pakistan; 2 Department of Computer Science, The University of Lahore, Sargodha Campus, Sargodha, 40100 Pakistan; 3 Heriot Watt University, Edinburgh, EH14 4AS UK; 4 Department of Computer Science and Information,, Applied College, Taibah University, Madinah, 42353 Saudi Arabia

**Keywords:** Urdu news, GloVe, True news, Fake news, UFND, Deep learning, Engineering, Mathematics and computing

## Abstract

Fake news has a significant effect on reader perceptions and is therefore a serious problem. It is difficult to differentiate between fake and real news as the number of News platforms on social media are growing daily. This work aims to create a complete fake news detection mechanism for Pakistani news by using many fact-checked APIs. A total of 14,178 real and fake news items from fifteen different regions and sections of reputable and authoritative Urdu newspapers and news broadcasting websites in Pakistan are included in the dataset. We evaluate the dataset via three deep learning models RoBERTa, XLM-RoBERTa and mBERT are embedded with concatenated BERT and GloVe. For the training of the selected models, we first use an extensive multilingual dataset. The results of the proposed models are as follows: evaluated via performance metrics such as the F1 score, accuracy, precision and recall. XLM-RoBERTa with concatenated GloVe embedding outperforms with an F1 score of 0.956, accuracy of 0.962, precision of 0.932 and recall of 0.940. A comparative analysis of the latest machine learning and deep learning models is also done. The latest Urdu benchmark dataset shows that the XLM-RoBERTa model with Concatenated BERT and GloVe embedding outperforms these models for Urdu fake news detection.

## Introduction

Owing to the rapid expansion of internet access over recent decades, its role in daily life has grown significantly. As a result, print media consumption has declined, while online news consumption continues to rise. Although information now spreads faster than ever, the credibility of online news remains a serious concern. Misleading information can influence public opinion, undermine trust in institutions, and affect a country’s political environment. Fake news has increased sharply on websites and social networks. Existing fact-checking platforms primarily focus on English content and offer limited support for regional languages. Effective fake news detection techniques rely on large and accurately annotated datasets. However, the absence of reliable lexical resources and the availability of only small-scale datasets hinder the development of natural language processing systems for regional and low-resource languages.

The Information revolution has ushered in far reaching changes in the lifestyle of the internet users. Fabricated and False information is spread online in the web and the Online social networks (OSNs). Burdened with fake news (FN), the users unwillingly distribute them in their social circles without checking the facts^1^. A reader’s mentality may be greatly affected by false information, which might cause them to make poor decisions. This can have a serious effect on health and finances^2^. Majority of the population are users of social media, they will share news on the social media sites, and most of the news is fabricated. The fake news (FN) mostly poses a threat to the most important spheres, i.e. politics, healthcare, religion, economy etc. and leads to social instability in the country^3^. Therefore, identifying erroneous information is essential. Fake news can be divided into three primary categories: fabricated, misleading, and partially genuine^4^. The most common technique for spotting fake news is human judgment; however, Sometimes, this method’s dependability may be undermined. A person must have a thorough comprehension of the relevant subject matter to correctly identify bogus news^5^. Therefore, It is essential to implement an automated system to detect fake news. NLP has been shown to be relevant in prediction evaluation in different ways. tasks related to language and gave fake news detection (FND) a new impetus. Moreover, transformer based models offered a possible remedy to fake news detection (FND) in resource-deprived languages^6^. The dataset is essential for any artificial intelligence system to automatically identify bogus news. LIAR^7^, Fake-or-Real news^8^, Twitter News^9^, and Weibo News^9^ uses a well-known dataset. There are a wide range of news items of topics in these databases, but none of them are focused on news from a particular region or country. Online data that have been scraped specifically for Pakistan are utilized in several ways to detect fraudulent information. No benchmark artificial intelligence method can be developed or evaluated with the minimum number of samples that are supplied. Therefore, A large dataset was used to detect Urdu fake news, with a particular emphasis on news from Pakistan.

**Fig. 1:**
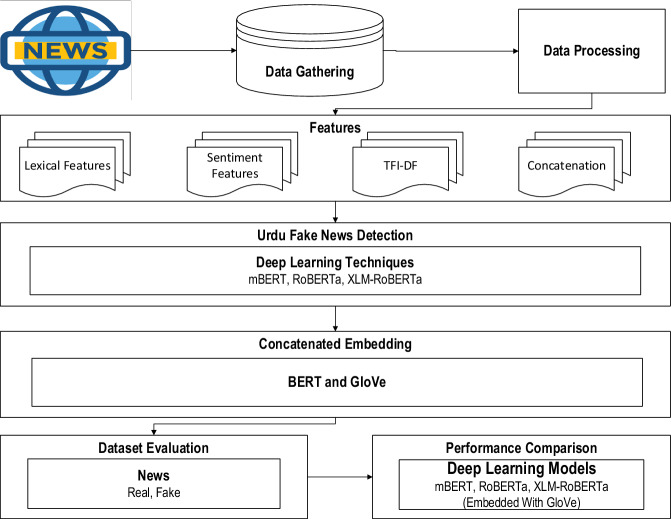
Proposed Urdu fake news detection methodology using deep learning.

The goal of this study is to compile an exhaustive dataset to identify Urdu fake news in the Pakistani media. The sophisticated deep learning technique such as GloVe embedding is used to assess the dataset and the effectiveness of many relevant deep learning approaches are evaluated and cross-compared. There are many performance measures, such as accuracy, precision, recall, and the F1-score, which form the foundation of the current study. The proposed methodology comprising of three sophisticated deep learning algorithms, mBERT, RoBERTa, and XLM-RoBERTa embedded with GloVe as depicted in Fig. 1. The main contributions of this work are as follows:

- Three deep learning models are used mBERT, RoBERTa, and XLM-RoBERTa for evaluating the dataset.
- The concatenated BERT and GloVe embedding is applied to improve the performance of existing models which effectively helps to evaluate Urdu fake news detection.
- The effectiveness of the proposed concatenated framework is tested using two real-world corpora with references to several DL approaches

The rest of this paper will be structured as follows: Section “Related work” presents a discussion of related work, whereas section “Material and methods” focuses on dataset evaluation, removing features, and applying deep learning models along with concatenated BERT and GloVE embedding. Section “Results and discussion” discusses the outcomes of the different models. Finally, section “Conclusion” provides the conclusion.

## Related work

This section provides an overview of the need for different approaches to identify fake news. It also identifies the limitations of the currently available datasets.

### Automated detection of fabricated news

The necessity for automated fake news detection has been highlighted in several fields. Recently, scholars have been keen to address the problem of fake news and how to spot it. The need to conduct research on automated fake news detection in several languages, specifically Asian languages, was emphasized in^10^. An automated Hindi FND based on twenty-four linguistic elements was used, and a recommended approach was established and they have Embedded words into HinFakeNews data using Hindi language peculiarities and achieved 98% accuracy in fake news detection (FND)^11^. To evaluate fake news (FN) in a rich-resource language, deep transfer learning methods of transformers were applied in English. The results of 92.88% accuracy and 92.89% F1-score established the superiority of the suggested method. zero and few-shot settings^12^. The study compared the efficacy of context free UFND embedded with Word2Vector embeddings that how its is effective in fake news detection. Their approach outperformed ML, DL, and SOTA algorithms in detecting spam across rich-resource language datasets contain multiple languages, excluding urdu. The ISOT Fake News dataset yielded the greatest classification accuracy 99.95%^13^. The multimodal approach implemented BERT and CNN and it showed good results. global enhancement of the classification results when compared to the old methods. The authors in the article by itself centered on content analysis and graph analysis with regard to FND^14^.

Moreover, automated methods were used to support the creators of the content and those who were consuming when determining the credibility of online news^8^. In addition, a systematic procedure to confirm information, approve content, and monitor Social media feeds were also proposed in^4^.

### Faking the news datasets

Several publicly available Urdu and English datasets are composed mainly of news stories related to politics. Data collected from online news sources are used to create models. Several researchers have used these datasets for English fake news detection, and few have used their translation skills to translate the English news datasets into Urdu for their research. The “Liar, Liar Pants on Fire” dataset is openly available and extensively used for the analysis and investigation of English fake news^15^. In this dataset, more than 12 thousand fake news statements were used which have been divided into 6 different categories. For fake news detection, the majority of researchers have used other datasets such as Weirdo, BuzzFeedNews, Twitter, and Fake-or-Real news^2, 4, 16, 17, 18, 19^. Several web-harvested datasets, including media news from Pakistan and other countries were also used to identify fake news^10^. There are also multiple Urdu fake news datasets (UFND) that are available, such as “The Bend the Truth” dataset, which includes 9 hundred news articles on Urdu^20^. News from five different domains were found in this study. However, this dataset includes only a limited number of samples. An extra 4 hundred news items were incorporated in the existing dataset, especially to test the data, to support the Urdu fake News detection (UFND) shared task in^21^. The dataset for UFND shared assignment includes a total of 3 hundred news articles in^22^. 4K Urdu news from ten different domains is included in the dataset^23^. One of the benchmarks dataset with 10K news items from 15 different domains is available on GitHub by^24^. In^27^, the efficacy of ML approaches for the fake news detection (FND) was evaluated via a benchmark dataset.

### Key features

Considerable efforts have been made to determine the key elements that may increase the accuracy of the models. Communication, discourse, and pragmatics have been used to achieve this goal in^25^. Elements such as slang, vulgarity, and the lexical and semantic qualities of a headline play a significant role as highlighted in^26^. The accuracy can be enhanced by supplementary data, such as a person’s social media interaction as depicted by^2^. Another important aspect, e.g., speaker characteristics, title, location, the party support, and the previous history of credit are also considered for fake news detection^9^. In^10^, the term of frequency and the term of inverse frequency are used for better detection of fake news.

### Urdu Fake News Detection (UFND) using ML and DL techniques

Fake news detection (FND) has increasingly popular over the last 20 years. In previous years many machine learning and deep learning techniques were used for the verification and authenticity of fake news. Research on Urdu fake news detection (UFND) has not been pursued extensively, and few studies have shown machine learning (ML) techniques applied for UFND. Furthermore, news about politics, religion, and other societal issues is not included in these datasets because these are delicate subjects that might incite people to inadvertently or intentionally spread misleading information on online social networks. Moreover, a range of phrases that may be used to find propagation patterns were provided by multidomain data. Therefore, when applied to small domain-specific datasets, the efficacy of UFND techniques are negligible in contrast to large datasets.

The Urdu fake news detection (UFND) dataset has been the subject of several studies^20, 21, 22^ using machine learning (ML), deep learning (DL), and natural language processing (NLP) models. FastTextfeatures and Term Frequency and the Term Inverse Document Frequency (TIDF) has been used in^22^. They achieved an accuracy of 0.79 on the test data. A variety of pretraining strategies, such as character-level convolutional neural networks (charCNN), the robust optimized BERT pretraining approach (RoBERTa), and label smoothing features, were also used in^28^ to obtain an accuracy of 0.91. In^29^, an F1-score of 0.817 and an accuracy of 0.82 has been achieved when a bidirectional GRU model to identify bogus news in Urdu. The problem of UFND was as follows: proposed in^30^ via an ensemble technique. Experiments were conducted on a freshly gathered dataset in addition to the dataset provided by^20^. There were 2000 news items in the new dataset, and no further domain information was included. The dataset includes the urdu news that were translated from english. In addition, the dataset^20^ did not undergo human verification and include only news articles from the five designated areas. The findings showed a decline in accuracy, and the available data were insufficient to produce a precise model.

A standard dataset for Pakistani fake news detection^31^ used numerous machine learning methods, e.g, decision tree ML algorithm, KNN ML algorithm, support vector machines ML algorithm, logistic regression ML algorithm, and naive Bayes ML algorithm. GloVe embedding and a BERT embeddings were also employed via the CNN and LSTM algorithms^31^. Nonetheless, the news articles were published in English. There are approximately 11,000 labeled news items in the collection. Two deep learning (DL) and five machine learning (ML) methods were also employed in the assessment. However, with GloVe embedding along with the LSTM, the model produced the highest F1-score of 0.943. It also shows that a large dataset outperforms a limited number of samples at the FND and yields superior results. 4K Urdu news articles in the dataset^23^ were collected from various sources. However, a significant portion of the sample’s local news items were translated from English sources. The accuracy of 0.93 was obtained by combining random forest algorithm with extra tree algorithm and logistic regression algorithm for dataset analysis. One of the researchers also employed an LSTM and RESNET model for Urdu fake news detection in^32^. Fake Urdu tweets were also detected in^33^ via the use of ML and DL techniques. They produced a dataset of 12K tweets and tested it via different CNN and RNN models^34^. It also provides a method for identifying fake news on websites and Twitter by utilizing the BERT methodology. The aforementioned datasets mainly focus on politics and contain tagged news data on a variety of topics. Owing to the small sample size and the frequency of fake news, the datasets are inappropriate to employ them to compare them to the artificial intelligence methods to identify fake news. Thus, there is an urgent demand to have all-inclusive data that can help in designing and testing algorithms that offer more precise findings.

**Fig. 2:**
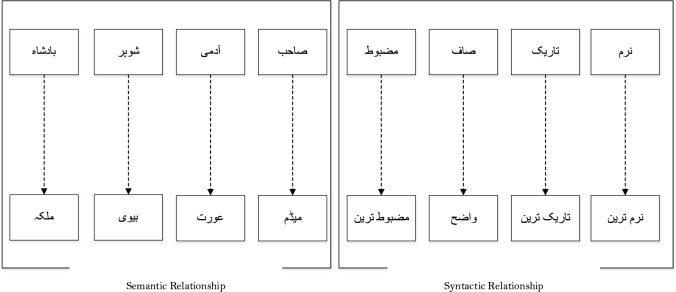
Semantic and syntactic relationship in Urdu text.

## Material and methods

**Fig. 3:**
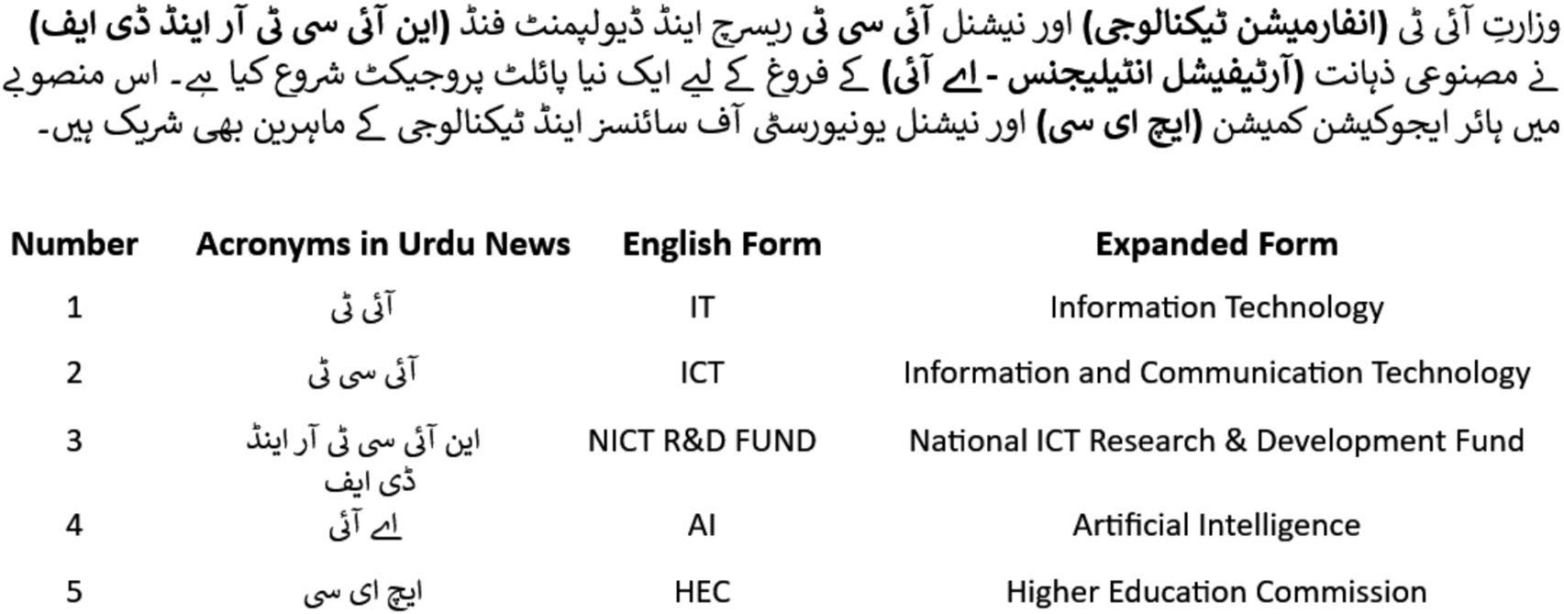
List of acronyms in Urdu news.

The dataset used in our research includes 14,178 news articles. The articles are divided into fifteen different categories. The dataset aims to address the shortcomings of earlier research in Urdu fake news detection (UFND). The news compiled between 2017 to 2023 includes a variety of fields such as sports, education, politics, health, business, technology, showbiz, science, crime, travel, enviornment etc. The keywords for searching Urdu-language news are Pakistan Urdu-language news, Punjab Urdu-language news, Balochistan Urdu-language news, Sindh-Urdu language news, KPK Urdu-language news, Azad Kashmir Urdu-language news, Gilgit Baltistan Urdu-language news, Pakistan and India Urdu-language news, PTI Urdu-language news, Pak Army Urdu-language news, N-League Urdu-language news and elections Urdu-language news, as mentioned in Table 1. The dataset looks at the patterns of deceptive narrative spread and included both cross-domain and multidomain news to increase lexical variety. Moreover, it evaluates the efficacy of many state-of-the-art models by an analysis of the terms used are shown in Fig. 2. The list of acronyms in Urdu news is shown in Fig. 3 .The contents are made up of news articles from reputable Pakistani Urdu news websites on national and international news topics.

**Table 1:** Search terms for gathering information about Pakistani news.

Regions and domains	Keywords
Pakistan	Pakistan Urdu-language News, Pakistan International Relations Urdu-language News
Balouchistan	Balouchistan Urdu-language News
Punjab	Punjab Urdu-language News
Sindh	Sindh Urdu-language News
KPK	KPK Urdu-language News
Azad Kashmir	Azad Kashmir Urdu-language News, Kashmir Urdu-language News
Gilgit Baltistan	Gilgit Baltistan Urdu-language News, Gilgit Urdu-language News
PTI Party	PTI Urdu-language News, Imran Khan Urdu-language News
Pak Army	Pak Army Urdu-language News, Army Urdu-language News. Pakistan Army Urdu-language News
N-League	N-League Urdu-language News
Election	Pakistan Election, PTI Election Results, N-League Election Results, PPP Election Results
Pakistan and India	Pak India Urdu-language News, Pakistan India Urdu-language News
Pakistan Economy	Pakistan Economy Urdu-language News
Pakistan Sports	Pakistan Sports Urdu-language News
Pakistan Social Issues	Pakistan Social Urdu News, Human Rights Urdu News, Pak Gender Issues Urdu News, Pakistan Education Urdu News

### UFND dataset collection

**Fig. 4:**
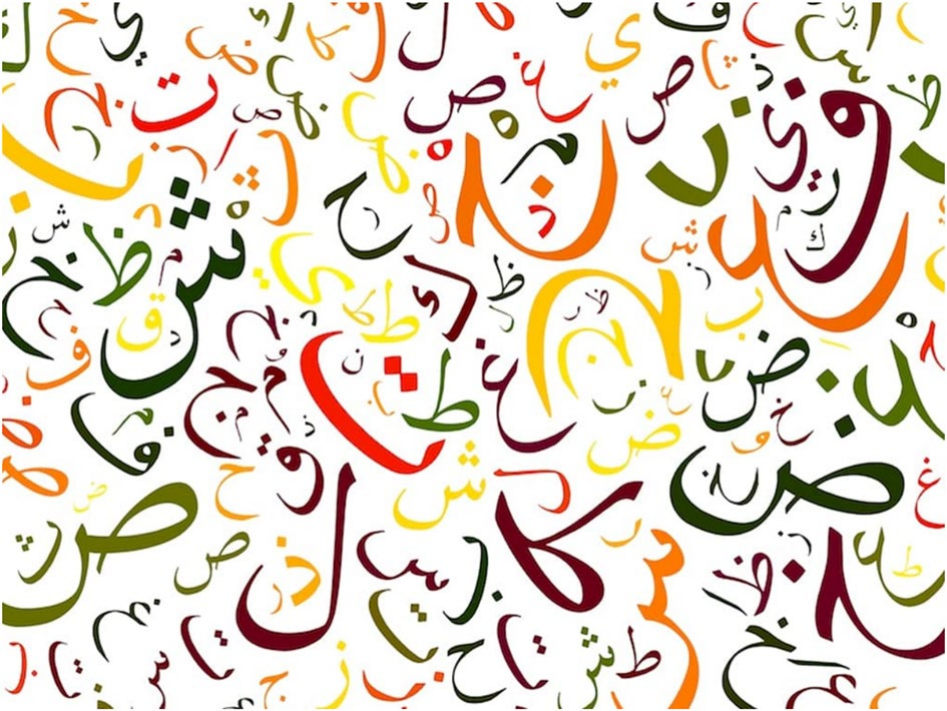
Urdu alphabets.

In this study, the first comprehensive dataset of fake news is used, which includes 14,178 instances of Urdu fake news related to Pakistan. Figure 4 illustrates the 36 letters used in the Urdu language. All words that are a combination of these letters appear in Urdu news.

#### Data compilation

By reviewing many sources and search for information about Pakistan, people often use keywords associated with Pakistan and its news. A list of keywords is combined to obtain news articles from Pakistan. Real and fake news articles are included in PolitiFact’s data collection, as this website saves application programming interfaces (APIs), which are specifically meant for obtaining and accessing news-related information. A popular and recognized representational state transfer (REST) API that adheres to user-friendly standards is the News API. It offers a wide range of authentic news from various international media sources. Only news content that is relevant to the specified category is collected via this API. The search word is entered into the API, which then uses it to search for news related to the “FactCheck” query. The nonprofit group FactCheck provides verified news from reliable sources as well as the reputations of false claims made by various politicians. A combination of real and fake news is compiled on the FactCheck website. Current datasets can be used to identify fake news in each of these topic areas, which includes news articles related to Pakistan in particular. News articles related to real and fake categories can be found in databases such as Urdu Point, Roznama, BBC, and Pak India News. These are social media platforms that offer both real and fake news in Urdu. We have compiled a selection of reliable and fake news from various other sources as well.

#### Challenges encountered in data collection

Compiling real and fake news is the process of gathering information for the fake news detection dataset. A number of issues complicate the data collection process. The news data must come from a trusted source to be considered legitimate, regardless of whether it is real or fake. Nevertheless, there are few trustworthy sources that provide information about fake news. Many news websites do not provide an API for gathering information. As a result, these websites’ news content has been removed because the data that is scraped just contains the HTML code of the loaded page and no real content. Few websites place limitations on data scraping in place, so the HTML content of these websites was gathered via page source access, and the data were then extracted from the hard-coded HTML content. Most fact-checking Websites provide credible international news; however, there is a lack of fact-checked news information regarding Pakistan. Most fact-checking websites lack a search function to find Urdu news and very few websites offer structured and well-defined REST APIs for sourcing news information. The majority of the websites do not have APIs or do not keep them up to date. PolitiFact is one such platform that maintains or offers a news collection of APIs. Since most fact-checking websites are international, they provide fact-checked news from all over the globe. However, the quantity of data received is much decreased when the fact-checked news data are limited to Pakistan. PolitiFact has carried out 170 fact-checks that are particularly related to Pakistan. This website has a large library of vetted news stories and is considered to be a well-known fact-checking source.

**Fig. 5:**
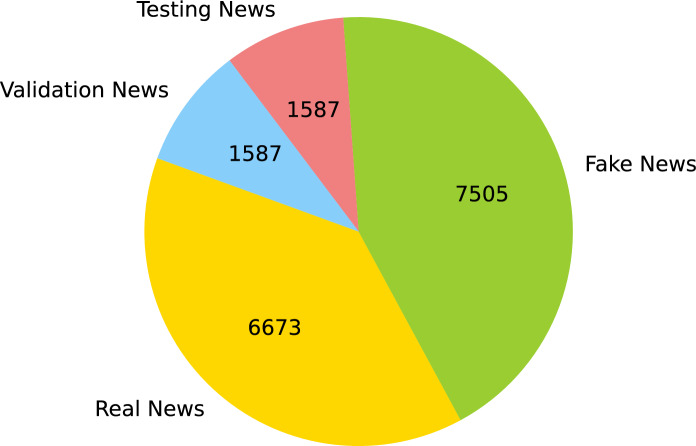
Urdu fake news detection (UFND) dataset statistics.

There is an imbalance in the class-related data as the problem of fake news identification involves binary classification, and can be classified as either authentic or fraudulent. The amount of accurate news data must outweigh the misleading news data according to the statistics gathered, indicating a major imbalance, as depicted in Fig. 5. Fact-checking resources are very limited, and few reputable Pakistani news websites are present that provide accurate and trustworthy news reports. Furthermore, the limited search scope of fact-checking websites results in a restricted quantity of information being provided.

**Fig. 6:**
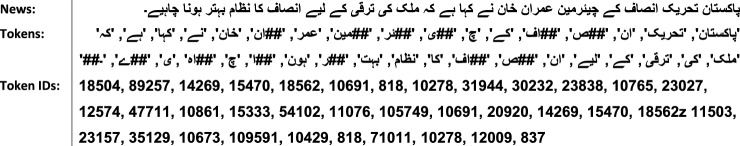
Tokenization process for Urdu text.

#### Data cleaning

This sub-section presents the process of inadequate, imprecise, or extraneous data identification. Afterwards, the data are cleaned by adopting the following data-cleaning process: (i) First, the duplicate items in the news dataset are eliminated; (ii) second, data purification is done by taking into account linguistic substance; (iii) third, metadata such as the URL, title, review date, publisher website, publisher name, content and author information is included in the gathered data; (iv) fourth, The news data that is collected from different sources is initially unrefined that will effect the process of fake news detection. Pre-processing is performed on the raw news text before it is fed into the models for fake news detection. The following pre-processing procedure is taken while organizing the news data set, Internet protocol addresses, Uniform resource locators (URL), and punctuation are removed, along with text segmentation into individual tokens. Furthermore, stop-words are removed, and stemming is executed as shown in Fig. 6; Last, the news report’s textual assessment labeling is performed, as the textual attribute has a range of values since the Urdu-language news are collected from different sources.

**Fig. 7:**
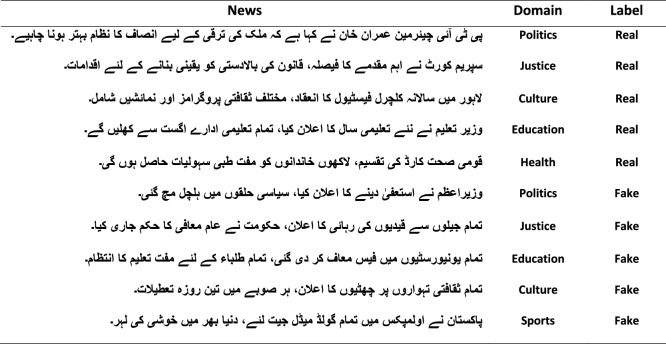
Fake and Real Urdu News along with their respective domains.

The textual assessments are divided into 15 categories for the identification of fake news. News labels are transformed into two different class labels for binary categorization. The false Urdu-language news have assigned to label of fake class; only the labels “accurate,” “partially accurate,” and “half accurate” are converted to the real class as shown in Fig. 7.

#### UFND dataset statistics

The dataset is complete after it has undergone cleaning and preprocessing. There are 14,178 labeled samples in the collection. The statistical data from the produced dataset is shown in Table 2, and the total number of real and fake news from different domains is shown in Table 3.

**Table 2:** Statistical distribution of the UFND dataset across training, validation, and testing.

Total news	Training news	Validation news	Testing news
14,178	11,004	1587	1587

**Table 3:** Total number of Urdu real and fake news from different domains.

Domain	No. of records	Real news	Fake news
Health Urdu-language News	1100	708	392
Business Urdu-language News	1557	823	734
Sports Urdu-language News	1869	1192	677
Technology Urdu-language News	1315	824	491
Showbiz Urdu-language News	1384	803	581
Politics Urdu-language News	2107	896	1211
Science Urdu-language News	1934	1055	879
Crime Urdu-language News	658	420	238
Travel Urdu-language News	784	446	338
Education Urdu-language News	773	518	255
Environment Urdu-language News	697	598	99
Total Urdu-language News	14178	8283	5895

### Data availability and ethical considerations

The datasets included in this study is publicly accessible and widely employed in Urdu fake news detection studies. All the Urdu news that are in dataset are collected from authentic Urdu news websites. The Urdu news dataset does not contain any personal or user confidential information. Before analysis, the dataset is cleaned from all types of sensitive information that could potentially reveal source details. The dataset is open-source and is used in compliance with their respective data usage policies and it can be accessed from https://zenodo.org/records/7773474 and https://github.com/Sheetal83/Ax-to-Grind-Urdu-Dataset.git.

### Assessment of datasets

The effectiveness of the used dataset is evaluated by classifying true or false news via several ML methods and DL methods. To check the authenticity of news first the real and fake labels are converted into 0 and 1 binary the fake news have assigned the value of 0 and real news have assigned the value of 1. The false Urdu-language news have assigned to label of fake; only the labels “accurate,” “partially accurate,” and “half accurate” are converted to the real.

### Wrenching out the features

The features included in the models have a large effect on their effectiveness. As a result, many traits, including lexical features such as common words, named entities, word length, and syllable count, are considered. Sentiment features, such as positive, negative, or neutral emotions in text and TF-IDF elements that indicate how frequently terms appear in the text, are gathered from the text and combined with deep learning methods. Furthermore, concatenated BERT and GloVe embeddings are also used in deep learning techniques where BERT is called contextual word embeddings, which identify the meaning of words from the context of other words in the sentence, while GloVe embedding are static word embeddings that define words as vectors on the basis of the relative frequencies of their occurrence in the whole corpus Fig. 9. to enhance overall effectiveness.

**Algorithm 1:**
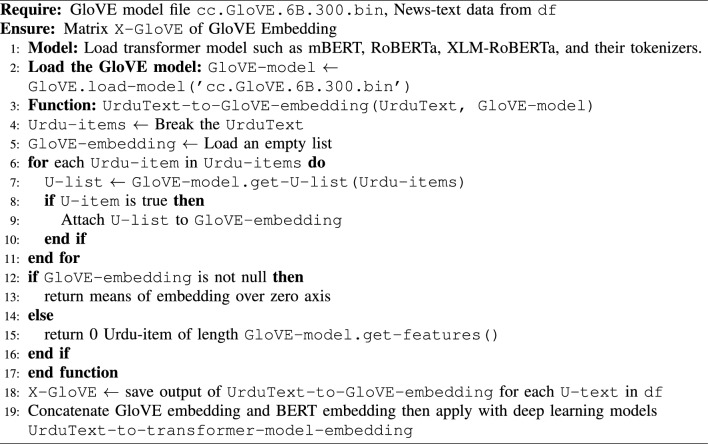
Deep learning models with GloVE Embedding for data in the form of text.

**Fig. 8:**
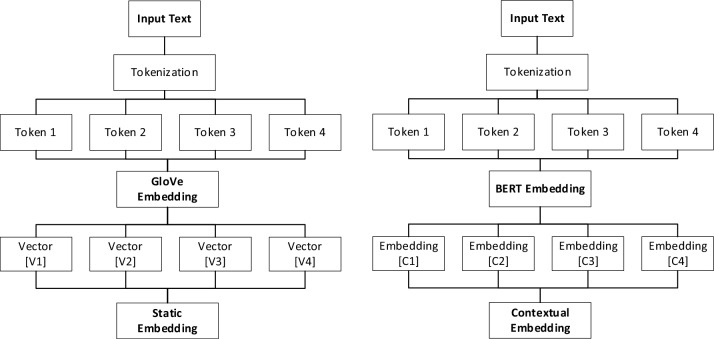
Workflow of GloVE and BERT embeddings.

### Deep learning approaches for UFND

To evaluate the dataset, several advanced deep-learning models along with concatenated BERT and GloVe embeddings have been used e.g., mBERT, RoBERTa and XLM-RoBERTa. The models are built with a learning rate of 1/1000 using the ADAM optimizer. Binary cross-entropy is used to create a loss function. The sigmoid function serves as the activation function for a single neuron that makes up the output layer. The training of models is done with the batch size of 64 across 30 epochs.

1 $$\begin{aligned} & \text {BCE}(a, b) = - \left( a \cdot \log (b) + (1 - a) \cdot \log (1 - b) \right) \end{aligned}$$

2 $$\begin{aligned} & \text {Sigmoid} \Sigma (a) = \frac{1}{1 + e^{-a}} \end{aligned}$$

3 $$\begin{aligned} & \text {ADAM } \theta _{(a+1)} = \theta _a - \frac{x}{\sqrt{\hat{w}_a} + \epsilon } \cdot \hat{n}_a \end{aligned}$$

The proposed method uses the GloVE embedding model to generate the embedding of words for the authenticity of news. The method Urdu text-to-GloVE-embeddings processes all texts and creates embeddings for each word via get-U-word-vector. This approach uses subword information to handle related words efficiently. These embeddings are averaged to reflect the entire text. If no comparable terms appear in the text, a zero vector is generated. When this approach is used in our dataset, it produces a feature matrix X-GloVE, which may be utilized for several analytical applications in our fake news investigation. Details of the GloVE embedding used in our paper is illustrated in Algorithm 1.

**Fig. 9:**
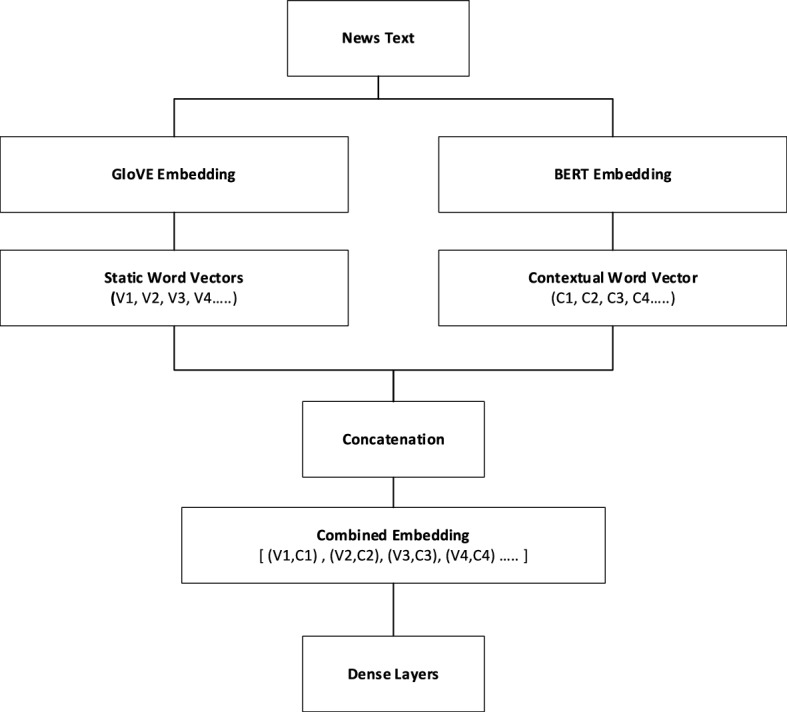
Workflow of concatenated GloVE and BERT embeddings.

**Fig. 10:**
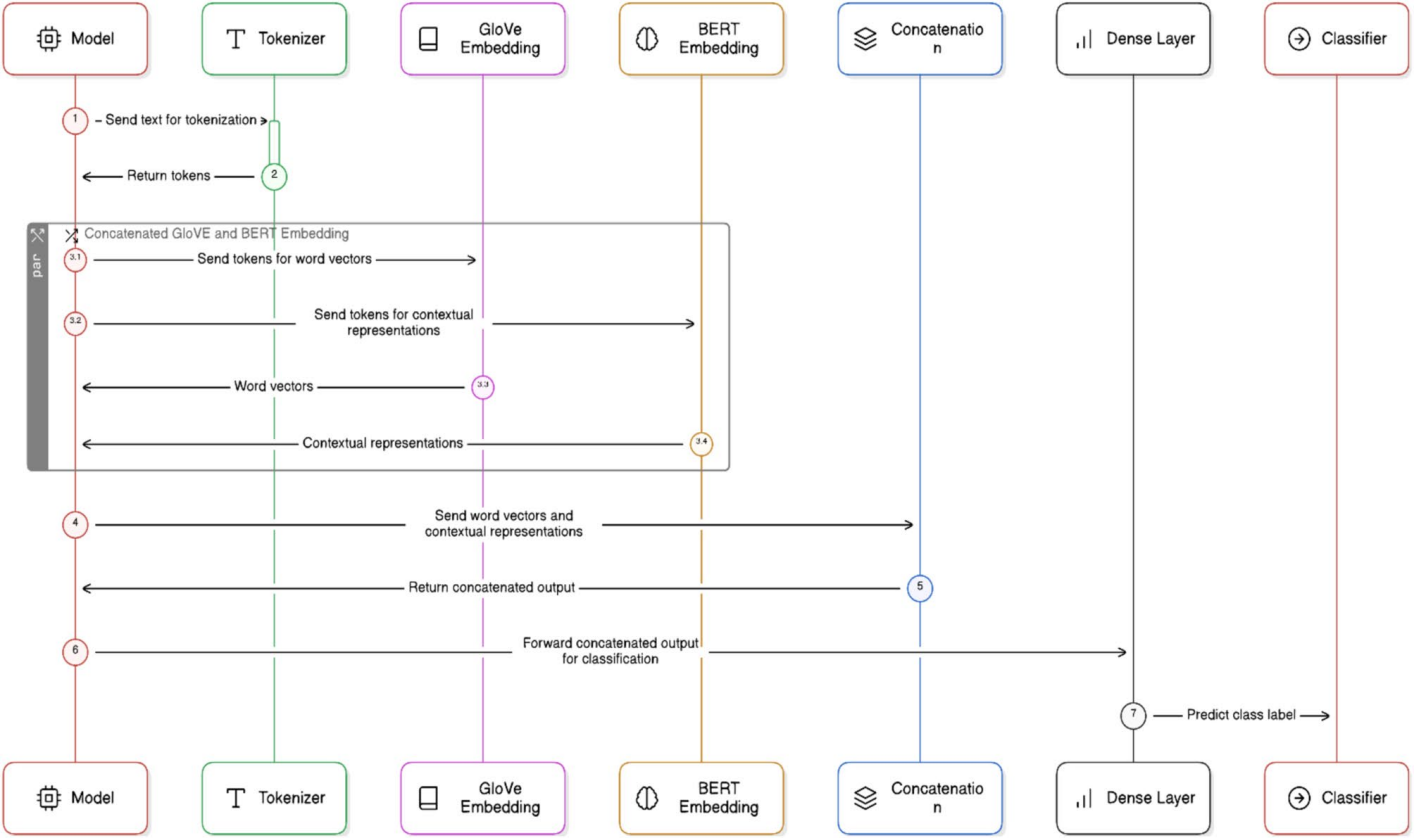
The concatenated BERT–GloVe embedding pipeline mechanism used for efficient model learning.

The GloVe word embeddings capture semantic similarities between words vectors as well as BERT used to generate contextual embeddings. These models will capture the contextual information from the news articles. The concatenated GloVe embeddings and the embeddings from the BERT model pass the entire article through mBERT, RoBERTa, and XLM-RoBERTa to get contextual embeddings Fig. 8. The pooling mechanism (e.g., mean, max, or attention-based pooling) is used to get a fixed-size vector representing the entire article. Afterwards, fully connected (dense) layers are used to process the concatenated embeddings. Figure 9. Finally, the sigmoid layer helps for binary classification of the data and the Adam optimizer helps to minimize the loss. The complete description of concatenated GloVe and BERT embedding that user input text the GloVE and BERT embedding generate Word vectors and contextual text for efficient learning of models is shown in Fig. 10.

## Results and discussion

In this section, a thorough examination of each model is along with a performance comparison. The pretrained models are trained on multilingual corpora making the enhanced performance clearly. The Models intricacy and model refinement increases its effectiveness for a particular classification task. Several metrics, including accuracy, precision, recall, and the F1-scores were compared and evaluated to determine the overall performance.

**Table 4:** Comparing the performance of several techniques using concatenated BERT and GloVe embedding.

Parameters	mBERT-WGE	RoBERTa-WGE	XLM-RoBERTa-WGE
Accuracy	0.938	0.946	0.962
F1-Score	0.930	0.938	0.956
Precision	0.918	0.926	0.932
Recall	0.924	0.932	0.940
P-values (vs XLM- RoBERTa-WGE)	0.032	0.041	-

Table 4 provides us the information about that how well the models performed via Concatenated BERT and GloVe embedding. mBERT performed well with an accuracy of 0.938 and an F1-score of 0.930. RoBERTa has an accuracy of 0.946 and the F1-score was 0.938. Finally, XLM-RoBERTa had an accuracy of 0.962 and an F1-score of 0.956.

### Performance validation

**Fig. 11:**
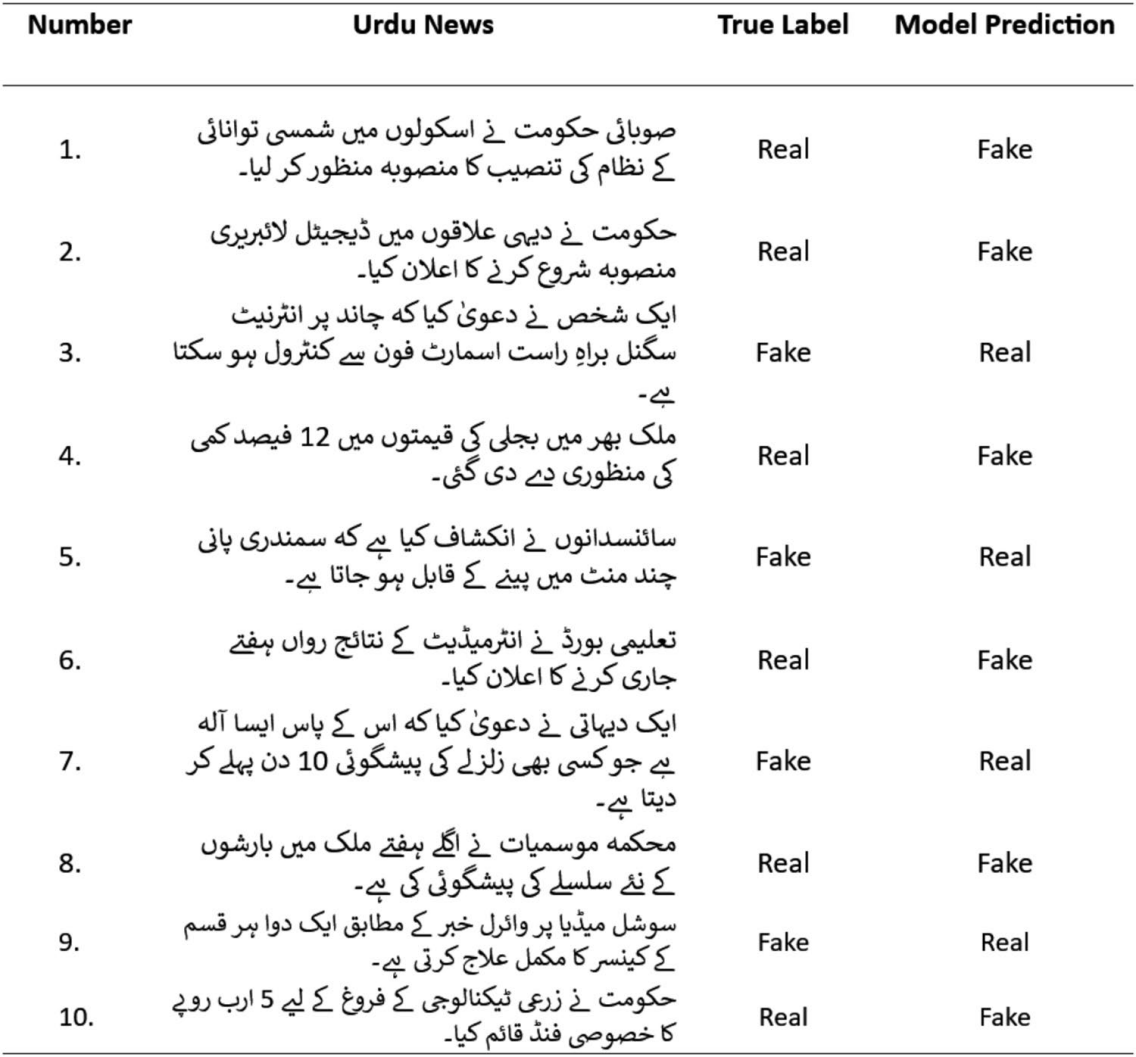
List of some misclassified Urdu news samples by XLM-RoBERTa-WGE.

The probability values (p-values) obtained from the Wilcoxon test indicate that the performance improvements of XLM-RoBERTa-WGE are statistically significant. The resulting p-values (mBERT:0.032, RoBERTa:0.041) are below the standard significance threshold of 0.05 ($p<0.05$). Examples of some misclassified Urdu news samples are shown in Fig. 11. Some of them are fake but were predicted as real, while some real news samples were predicted as fake. The content and meaning of the news play an important role in detecting the correct class.

Since NLP data such as news text often deviate from its normal distribution, so the Wilcoxon test provides more reliable information that performance improvements are not due to random chance, XLM-RoBERTa-WGE gain superiority over other models. The XLM-RoBERTa model with concatenated embedding outperformed all the other methods mentioned models. It is deduced from the results that XLM-RoBERTa is better than both mBERT and RoBERTa because it has a well-structured architecture conceived for cross-lingual purposes. The result also implies that concatenated embedding improves the performance of models to detect the real and fake news in UFND. A comparative analysis of well known deep learning techniques is also shown in Table 5. The UFND dataset shows that the XLM-RoBERTa model outperforms other state-of the-art deep-learning models. Furthermore, compared with machine learning and deep learning models, the mentioned individual pretrained transformer-based models performed better.

**Fig. 12:**
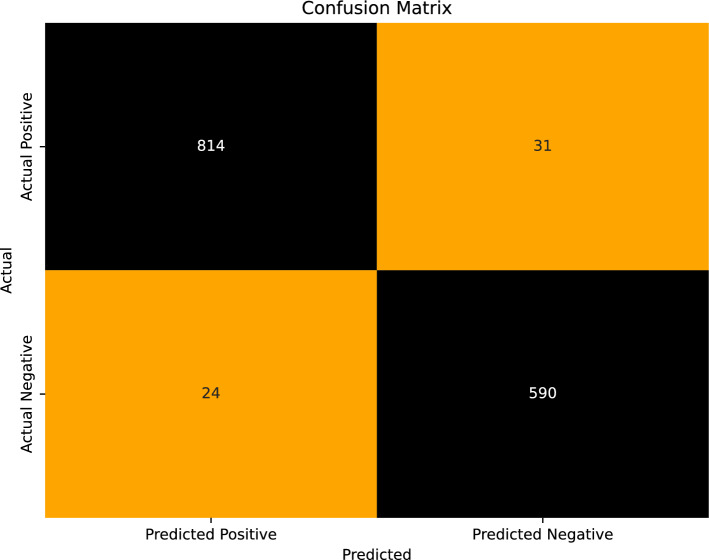
Confusion matrix using TFI-DF.

**Fig. 13:**
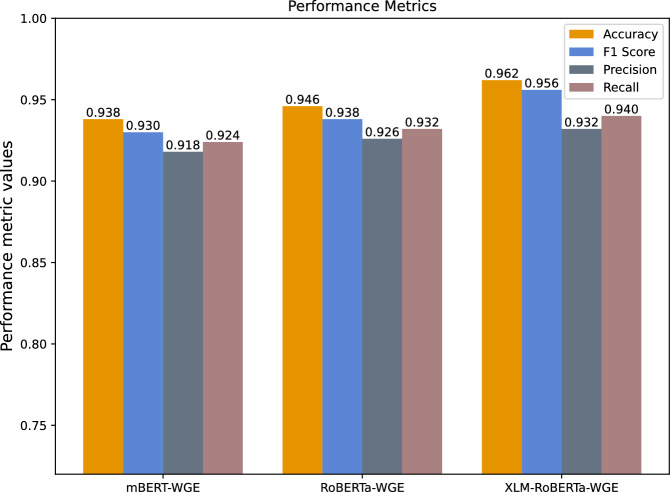
Comparison of various DL models with concatenated BERT and GloVe embedding technique.

Finally, the XLM-RoBERTa model with concatenated embedding from each model performs better overall. Therefore, it can be concluded that the proposed model works well for Urdu fake news detection (UFND). Figure 12 shows the confusion matrix with values of TP, TN, FP, and FNs on the 1587 UFN testing dataset. In Fig. 13, the model results are compared and shows that the performance of the XLM-RoBERTa model with concatenated BERT and GloVe embedding is better than all other ML and DL models. The comparison validates that combining the results of the selected models via GloVe embedding technique is reasonable. Furthermore, the overfitting issue is well mitigated by the proposed technique.

### Limitations and future directions

The proposed models are efficient for Urdu fake news detection (UFND) but are limited to text-based analysis. The dataset used in this study mainly contain the text-based news in Urdu language which prevents the models from working effectively. This research could be extended in the future by using the news having combination of contextual information features based on text, visual and social information from online platforms. We can enhance performance if the models are trained in multiple languages. For deeper understanding and reasoning, in the future, Large Language Models (LLMs) such as GPT-4, LLaMA-3, and Mistral could be used. The incorporation of these Large Language Models (LLMs) could further improve transparency in the detection of fake news.

**Table 5:** Comparing the performance of several techniques for Urdu Fake Detection UFND.

Existing approaches	UFN Count	F1-Score	Accuracy	Precision	Recall
amjad2020bend^20^	900	0.901	0.880	-	-
amjad2020urdufake^21^	1300	0.902	0.909	0.919	0.937
akhter2021sup^30^	1032	0.827	0.812	-	-
amjad2022^22^	1600	0.680	0.757	0.758	0.930
farooq2023fake^23^	4097	0.933	0.939	0.890	0.863
munir2024bil^32^	-	0.920	-	-	-
Proposed methodology	14,178	0.952	0.962	0.932	0,940

## Conclusion

The rapid proliferation of digital media and online social networks has significantly accelerated the dissemination of fake news, resulting in widespread user deception and societal unrest. This issue is particularly pronounced for low-resource languages such as Urdu, where limited linguistic resources and scarce high-quality datasets have constrained research progress. To address these challenges, this study proposed a comprehensive approach for Urdu fake news detection by leveraging state-of-the-art pre-trained language models and enriched feature representations. For experimental evaluation, the publicly available UFND dataset, consisting of 14,178 real and fake Urdu news articles, was utilized. The dataset encompasses news from credible Pakistani news outlets, newspapers, and online platforms across multiple domains, thereby enhancing lexical diversity and enabling effective analysis of misinformation. To capture rich semantic representations, pre-trained transformer models including mBERT, RoBERTa, and XLM-RoBERTa were employed, with feature representations further strengthened through the concatenation of contextual BERT and GloVe embedding. Extensive experiments demonstrated that XLM-RoBERTa consistently outperformed mBERT and RoBERTa across all evaluation metrics, achieving a maximum accuracy of 96%, while mBERT and RoBERTa achieved accuracies of 93% and 94%, respectively. These results highlight the effectiveness of combining multilingual pretraining, fine-tuning, and enriched embedding representations for Urdu fake news detection (UFND). The proposed framework is robust, scalable, and well-suited for tackling misinformation challenges in resource-constrained languages. Future research may explore more advanced transformer-based architectures and large language models (LLMs), such as Google Gemini, GPT-4/4o, and LLaMA-family models, to address complex scenarios including multi-label classification and real-time misinformation detection. Incorporating adversarial training techniques may also enhance model robustness against evolving and sophisticated misinformation strategies.

## Data Availability

The datasets included in this study is publicly accessible and widely employed in Urdu fake news detection studies. All the Urdu news that are in dataset are collected from authentic Urdu news websites. The Urdu news dataset does not contain any personal or user confidential information. Before analysis, the dataset is cleaned from all types of sensitive information that could potentially reveal source details. The dataset is open-source and is used in compliance with their respective data usage policies and it can be accessed from https://github.com/Sheetal83/Ax-to-Grind-Urdu-Dataset.git and https://zenodo.org/records/7773474.

## Abbreviations

ADAM: Adaptive moment estimation
BCE: Binary cross-entropy
BERT: Bidirectional encoder representations from transformers
CNN: Convolutional neural network
DL: Deep learning
FND: Fake news detection
GloVe: Global vectors for word representation
KNN: K-Nearest neighbors
LSTM: Long short-term memory
LLM: Large language model
NLP: Natural language processing
API: Application programming interface
RoBERTa: Robustly optimized BERT approach
TF-IDF: Term frequency–inverse document frequency
UFND: Urdu fake news detection
WGE: With GloVe embedding
ML: Machine learning
XLM: Cross-lingual language model
